# The Long-Term Effective Rate of Different Branches of Idiopathic Trigeminal Neuralgia After Single Radiofrequency Thermocoagulation: A Cohort Study

**DOI:** 10.1097/MD.0000000000001994

**Published:** 2015-11-13

**Authors:** Yuan-Zhang Tang, Bai-Shan Wu, Li-Qiang Yang, Jian-Ning Yue, Liang-Liang He, Na Li, Jia-Xiang Ni

**Affiliations:** From the Department of Pain Management, Xuanwu Hospital of Capital Medical University, Beijing, China.

## Abstract

To evaluate the efficacy of computed tomography (CT) guided single radiofrequency thermocoagualtion (RFT) in 1137 patients with idiopathic trigeminal neuralgia after a follow-up period of 11 years, specially focused on duration of pain relief in different branches of trigeminal nerve, side effect, and complications.

Retrospective study of patients with idiopathic trigeminal neuralgia treated with a single CT guided RFT procedure between January 2002 and December 2013.

The mean follow-up time was 46.14 ± 30.91 months. Immediate postprocedure pain relief was 98.4%. V2 division obtained the best pain relief rate: 91%, 89%, 80%, 72%, 60%, and 54% at 1, 3, 5, 7, 9, and 11 years, respectively. No statistical difference pairwise comparison was in other groups. The complications included masseter muscle weakness, corneitis, diplopia, ptosis, hearing loss, limited mouth opening, and low pressure headache. Masticatory weakness mostly occurred in patients with V3 branch involvement, while Corneitis and Diplopia all in patients with V1 branch involvement. No mortalities observed during or after RFT.

All different branches division of trigeminal neuralgia achieved comparable satisfactory curative effect; V2 obtained the best excellent pain relief, after RFT procedure. Facial numbness is inevitable after RFT, which patients who have pain in all 3 trigeminal divisions and patients who desire no facial numbness should be cautious. Masticatory weakness is mainly related with V3 injured, while Corneitis and Diplopia in patients with V1 injured by RFT.

## INTRODUCTION

Idiopathic trigeminal neuralgia (ITN) is presented as excruciating and causes serious impairments of the quality of life, with mean annual incidence of 12.6 per 100,000 person years.^1^ It is the most common painful condition characterized by nondemonstrable structural lesion, which is different to symptomatic trigeminal neuralgia (STN). Fortunately, regardless the mechanism of ITN is currently not well known, various effective treatment modalities have emerged, and treatment guideline has been developed.^2–4^ Treatment options for ITN include medications and interventional procedures. First-line treatment should be medications, including anticonvulsant drugs, muscle relax, and neuroleptic agents. For patients who cannot tolerate the medications or with intractable pain, several neuro-surgical procedures (ablative gasserian ganglion percutaneous techniques, gamma knife, and microvascular decompression [MVD]) are available to treat ITN. Notably, each procedure has advantages and disadvantages, prompting the question as to which is most suitable for individual patients.

MVD, although effective, is the most invasive and has a mortality rate of 0.2% to 0.5%. This makes it unsuitable for many elderly patients, and for others who are considered as poor surgical risks.^4,5^ For such patients, ablative neurosurgical techniques may be used. Radiofrequency thermocoagulation (RFT) is reported to give higher rates of complete pain relief than either glycerol rhizolysis or stereotactic radio surgery.^6,7^ The procedure success rate of RFT approaches 100%, which is superior to that of MVD, which is only 85%. However, its long-term efficacy is lower.^6^ However, as well as being safe in elderly patients, and in those who are poor surgical risks, it has the advantage that it can be repeated, if required.^8^ Taha and Tew^7^ have proposed that neurosurgeons should gain proficiency in different techniques, so as to be able to offer the most suitable procedure for individual patients; MVD only being recommended for otherwise healthy patients with isolated pain in the distribution of the first division or of all 3 divisions of the trigeminal nerve, and in patients who desire no sensory deficit. However, Effective Rate of Different Branches on ITN is poorly known.

In this study, we report a retrospective study analyzing the outcome in patients with ITN treated with a single RFT procedure. Especially focus was placed on the duration of pain relief of Different Branches of trigeminal nerve, side effect, and complications.

## MATERIALS AND METHODS

### Patients

Patients were eligible for inclusion if they had undergone RFT treatment for ITN in the Pain Department at our hospital between January 2002 and December 2013. ITN was diagnosed using the criteria of the International Classification of Headache Disorders-II (2004). The patients, if the duration of their symptoms was <2 years, or the characteristics of the pain had changed in the preceding 6 months, had to receive a Cranial magnetic resonance imaging (MRI) to exclude multiple sclerosis, carcinomatous pain, or other causes of STN. We obtained follow-up information on patients by telephone interviews and from their medical records. If patients had died, information was obtained from their relatives. If neither the patient nor a relative could be traced for telephone interview, the patients were considered as lost to follow-up. The study was approved by the local ethics committee. Informed written consent was obtained from all patients.

### RFT Procedures

Our RFT procedure has been reported previously in detail.^8,9^ After the RFT procedure, patients were put on strict bed rest for 2 days, to prevent intracranial hypotension (low pressure) headache. They were usually discharged from hospital on the 3rd postprocedure day. Carbamazepine, previously prescribed for pain control, was gradually weaned over a period of 1 week, postprocedure, to prevent withdrawal symptoms.

### Data Collection

Demographic, clinic, and RFT data were all recorded. With regard to the complications, particular attention was paid to reports of masseter weakness, corneal inflammation, hearing loss, ptosis, and difficulty opening the mouth.

According to the different branches division of trigeminal nerve, all patients were divided in 6 groups: V1 (ophthalmic division) group, V2 (maxillary division) group, V3 (mandibular division) group, V1 + V2 group, V2 + V3 group, and V1 + V2 + V3 group.

Methods of pain intensity evaluation, pain-relief (“excellent,” “good,” “fair,” “poor”) and facial numbness/dysesthesia (grades I to IV) classification according to our previous reported.^9^

### Statistics

Kaplan–Meier survival curves and log-rank test applied in the evaluation of pain-free and dysesthesia-free after RFT. The censor point was: the last contact, the time of any subsequent surgery, or death. For statistical purposes, in patients who died, the duration of pain relief was assessed as the time from the procedure to the time of death. If a second RFT procedure on the same nerve during the same hospital stay was required, this was considered as a single procedure. If bilateral RFT procedures were undertaken on the same patient, this was treated as 2 procedures. All analyses were performed using SPSS for windows version of 17.0 (Chicago, IL). A *P* value of <0.05 was considered statistically significant.

## RESULTS

The 1113 patients underwent unilateral RFT and 24 patients underwent bilateral RFT, for a total of 1161 single RFT in 1137 patients; 121(10.6%) patients lost to follow-up evaluation and postoperative pain relief or complications did not significantly predict loss to follow-up. The mean postoperative duration was 46.14 ± 30.91 months. According the original pain site, 42 (3.1%) only with V1 trigeminal neuralgia were enrolled in V1 group, 289 (21.2%) in V2 group, 288 (21.1%) in V3 group, 66 (4.8%) in V1 + V2 group, 381 (27.9%) in V2 + V3 group, 89 (6.5%) in V1 + V2 + V3 group, and 6 (0.4%) in V1 + V3 group. So, the highest incidence rate of distribution of trigeminal nerve is V2 + V3, and then V2, V3, V1 + V2 + V3, V1 + V2, the V1 division, and V1 + V3 division of pain alone is the lowest (Table 1).

**TABLE 1:**
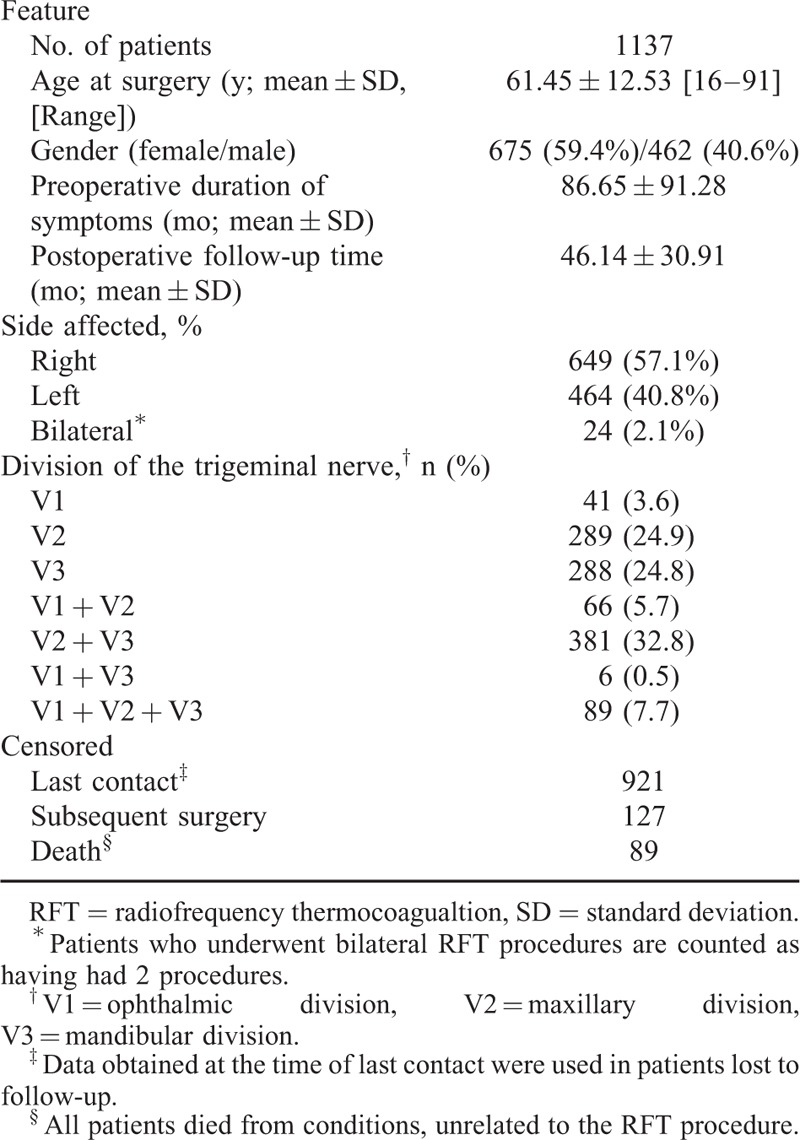
Demographic, Clinical, and RFT Data on Patients Studied

### Efficacy in Pain Relief and Pain Recurrence

**FIGURE 1:**
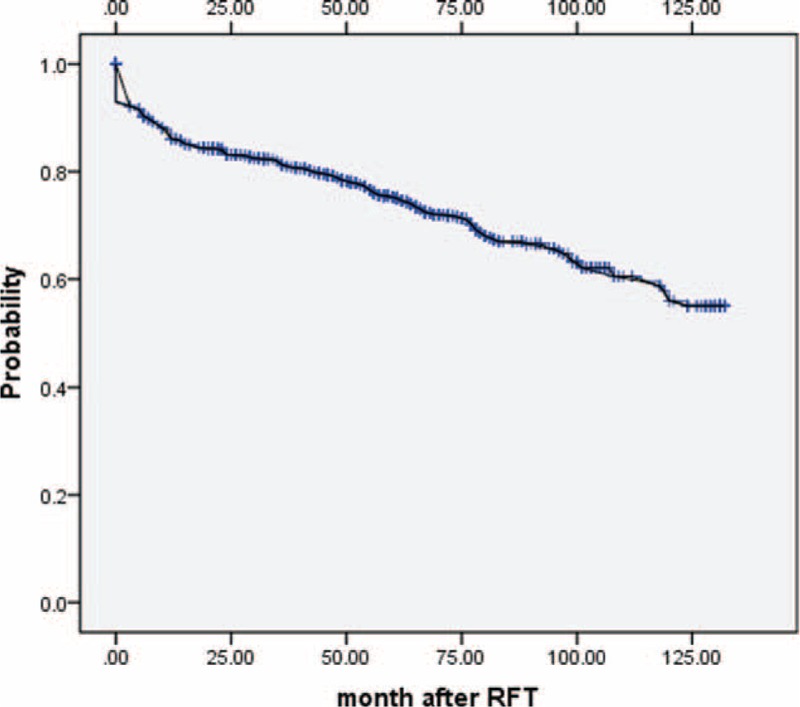
Acurarial Kaplan–Meier curve shows the long-term outcomes of patients pain-free off medications after radiofrequency thermocoagualtion.

**FIGURE 2:**
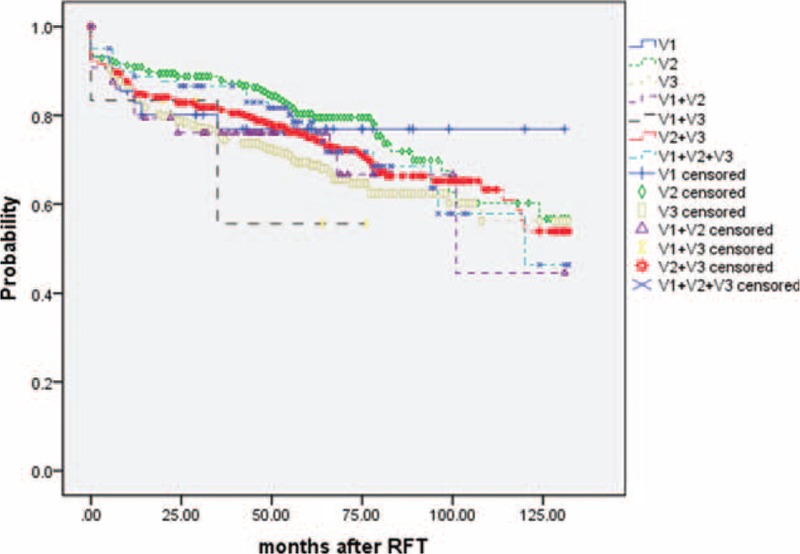
Acurarial Kaplan–Meier curve comparing the long-term outcomes of different branches of trigeminal nerve in ITN patients with pain-free off medications after radiofrequency thermocoagualtion. V1 = ophthalmic division, V2 = maxillary division, V3 = mandibular division.

Excellent pain relief was achieved 88% at 1 year, 79% at 3 year, 72% at 5 year, 65% at 7 year, 57% at 9 years, and 52% at 11 years (Figure 1). Immediate pain relief outcomes (excellent) at the time of hospital discharge were 1143 (98.4%) patients, and this finding was accepted as the initial success rate of single RFT. The excellent pain relief rates of the different branches division show in Table 2. We assessed the excellent pain relief data with a Kaplan–Meier actuarial curve (Figure 2), the log-rank test (for each stratum) showed the long-term excellent pain relief rate of V2, significant better than V3, V1 + V2, V2 + V3 (*P* < 0.05), the log-rank test (for each stratum) did not show any statistical difference pairwise comparison in other groups.

**TABLE 2:**
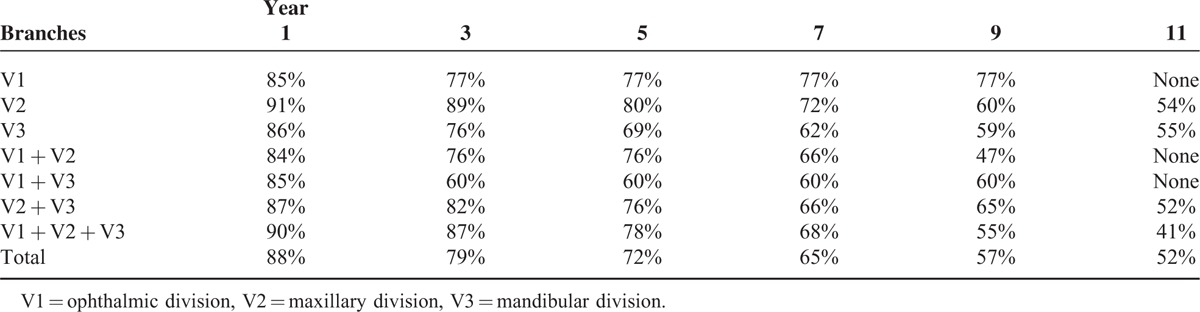
Excellent Pain Relief (No Pain, No Medications) Rate of the Different Branches Within 11 Years Follow-Up

The pain recurrence was observed in 9 (22% of 42 patients) patients within V1 distribution, 31 (10.8% of 289 patients) patients within V2 distribution, 51 (18.1% of 288 patients) patients within V3 distribution, 15 (24.2% of 66 patients) patients within V1 + V2 distribution, 0 (0% of 6 patients) patients within V1 + V3 distribution, 64 (17.2 % of 381 patients) patients within V2 + V3 distribution, and 16 (18.4% of 89 patients) patients within V1 + V2 + V3 distribution at after operation, for a total of 186 (16% of 1161 patients) patients had pain recurrence: 51 (4.4%) patients had medical treatment to control pain satisfactory; 135 (11.6%) patients underwent additional surgery, including repeat RFT (66); stereotactic radiosurgery (19); MVD (38); and percutaneous microballoon compression (12). Of 135, 14 (10.4%) did not achieve pain relief even after the additional surgery.

### Complications

The 983 patients (84.7%) had different degrees of facial numbness immediately after RFT; 57 patients have no facial numbness and 243 patients experienced facial numbness disappeared gradually with mean duration of follow-up of 31.4 ± 14.9 weeks; however, the complete pain relief of these patients was (167/300, 55.7%) lower than facial numbness patients (504/740, 68.1%). Seven hundred forty patients (63.7%) complained different degrees of facial numbness in the follow-up time, grade II in 398 (53.8%), grade III in 289 (39.1%), and grade IV in 53 (7.2%) patients. For the grade IV (painful dysesthesia), the incidence rate of V2 group is 1% (3 of 289 patients), V3 group is 9% (26 of 288 patients), V2 + V3 group is 3.7% (14 of 381 group), and V1 + V2 + V3 group is 11.2% (10 of 89 patients). Notably, all the patients said the facial numbness gradually reduced after the procedure.

Other complications include: masseter weakness was observed in 91 (8%) patients, especially 87 patients with V3 involvement (96% of 91 patients); however, all of them improved during follow-up; corneitis was observed in 29 (2.6%) immediately after procedure, all of them with V1 involvement, they were all treated with artificial tears to improve symptom, unfortunately, 1 patient ignored our advice and lose her right eye sight eventually; 14 (1.2%) patients suffered from Diplopia, all of them with V1 involvement, 2 patients were left with permanent diplopia induced by abducens nerve palsy; 2 patients had cerebrospinal fluid leak and suffered from intracranial hypotension headache because they get up within 2 days; these 2 patients improved by rapid infusion and lie in bed absolutely 1 week. Another complications included dropping eyelid (8, 0.7%), limited mouth opening (5, 0.4%) patients, and hearing loss (5, 0.4%). All these patients improved during the follow-up. We did not experience an occurrence of cranial nerve paralysis, carotid-cavernous fistula. There were no mortality and no permanent cranial nerve deficit except dysesthesia.

## DISSCUSSION

Nowadays, there are mainly 2 treatment options for trigeminal neuralgia (TN): ablative neurosurgical techniques (include radiofrequency thermocoagulation, glycerol rhizolysis, and stereotactic radiosurgery) and MVD. There are not any well-established guidelines on the choice of surgical intervention, each technique possess certain attributes and limitations. RFT of the trigeminal gasserian ganglion has been popularized since 1974, as an effective treatment which is less invasive than some alternatives.^10^ The heat produced by the RPF needle is thought to selectively destroy the Aδ and C pain fibers by thermocoagulation at temperatures above 65°C.^11^ This can prevent both triggering of pain and the intensity of pain, but can also cause numbness and other problems.^6,12^ A systematic review of ablative neurosurgical techniques for the treatment of trigeminal neuralgia found that when compared to glycerol rhizolysis and stereotactic radiosurgery, RFT achieves higher rates of complete pain relief, but is associated with more complications.^6^ MVD, which removes vascular compression at the root of the trigeminal nerve, is also reported to have a high success rate. Some^13,14^ surgeons consider that it should be preferred to RFT, as it provides pain relief without purposely damaging the gasserian ganglion, thereby avoiding numbness and dysesthesia. However, it is the most invasive technique, involving exploration of the posterior fossa, and when compared to ablative procedures, has a lower success rate.^7^ Moreover, it is not tolerated by some patients, especially those who are tomophobia and/or have multiple medical problems. It is associated with significant risks, including of mortality. A nationwide study of 3 invasive treatments for trigeminal neuralgia: partial sensory rhizotomy; RFT; and MVD, concluded that, although the likelihood of a repeat procedure being required was less with partial sensory rhizotomy and MVD, when compared to RFT, they were associated with a higher risk of complications requiring hospital readmission.^13^ Percutaneous techniques have been proposed as safer and more effective alternatives. A number of authorities restrict the use of MVD for treatment of trigeminal neuralgia to younger patients,^15^ and prefer RFT for older patients and/or those who are in poor health.^4,16^ Although we maintain some flexibility, our practice is to use RFT to treat those over 50 years of age, those considered to be poor surgical risks, and those who wish to avoid a craniotomy.

In our retrospective study, excellent pain relief in all patients was 88% at 1 year, 79% at 3 year, 72% at 5 year, 65% at 7 year, 57% at 9 years, and 52% at 11 years. A previous review of the treatment option in TN reported the success rate of pain free was 68% to 85% at 1 year, 54% to 64% at 3 years, and 50% at 5 years after RFT.^4,16^ The longest follow-up (20 years) outcomes after RFT reported the complete relief rate was 57.7%, 52.3%, 42.2%, and 41.0% at 5, 10, 15, and 20 years, respectively, despite lack of data within 5 years.^4,17^ As we know, the pain relief rate of RFT of different branches division of TN has not been reported before. In our study, we provide the most detailed data after RFT within 11 years follow-up. Each branches division underwent RFT obtained a good curative effect, they are in agreement with those of previous studies. However, the V2 branches division of pain alone achieved better “excellent” pain relief than other division, which indicate V2 branches has higher curative effect underwent RFT. Moreover, our treatment outcome in our patients receiving RFT was better than previous reports.

A possible explanation of our better treatment outcomes was that we used computed tomography (CT), rather than X-ray fluoroscopic, guidance to enable the cannula to be passed through the foramen ovale to access the gasserian ganglion, which is located on the floor of the middle cranial fossa. X-ray fluoroscopic guidance improves the ability to locate the ganglion, but with it there remains an appreciable failure rate, reported to be between 4.^18,19^ With X-ray fluoroscopy miss-insertion into the foramen spinosum, jugular foramen, inferior orbital fissure, or internal carotid artery are appreciable risks. The better visualization of bone and soft tissue with CT minimizes this. CT guided gasserian ganglion puncture is considered safe and effective.^20^ In our study, we correctly located the gasserian ganglion in all patients. Our findings support CT over X-ray fluoroscopic guidance.

Modern medicine seeks to develop more efficacious, less invasive surgical treatments, with quicker recovery and fewer complications for the patient. RFT satisfies the first 3 of these aims. A relatively high incidence of adverse effects is its principle drawback, limiting its more widespread use.

Postoperative facial numbness is common following RFT. It usually accompanies the relief from pain. Temperatures of over 65°C are known to destroy nerve fibers, with the Aδ and C fibers nociceptive fibers, being destroyed at lower temperatures than are required to destroy the Aα and Aβ tactile fibers. So we choose 65 to 85°C as our radiofrequency temperature in procedure. Usually, V1 branches division choose 65 to 70°C, while V2 and V3 choose higher temperature (75–85°C) for avoiding cornea and trochlear nerve damage. Generally temperatures of between 55 and 85°C are used during RFT to treat trigeminal neuralgia.^17,21^ Our radiofrequency temperature was agree with their reported. Facial numbness or painful dysesthesia is a serious side effect of RFT that affects the quality of life. For this reason, we further study the painful dysesthesia occurred in different branches division. We found V1 + V2 + V3 and V3 of pain alone have the higher incidence rate of painful dysesthesia and it mainly occurred in patients with V3 involvement. We inferred this maybe related with higher temperature on V3 branch. The optimal RFT temperature requires further study. Our study showed facial numbness occurs in a high percentage of patients undergoing RFT procedure; fortunately, facial numbness of most patients after RFT is mild, limited, and well tolerated. However, patients with V1 + V2 + V3 branches involvement usually have numbness in the entire face, this is not well tolerated. Therefore, we recommend RFT for patients who have pain in all 3 trigeminal divisions and patients who desire no facial numbness should be cautious.

Our rates for complications including masseter muscle weakness, corneitis, low pressure headaches, diplopia, hearing loss, ptosis, and limitation of mouth opening are similar to other published series of RFT to treat trigeminal neuralgia.^5,17^ These complications were all transient, except 1 patient who lost vision from corneitis, and another 2 patients with permanent diplopia. We found that masticatory weakness mostly occurred in patients with V3 branch involvement (96%). Masseteric nerve and masseteric nerve (originated from the V3 branch) were injured by radiofrequency is the main reason. Our previous study^22^ clarified that a high rate of masticatory weakness using surface electromyographic activity examination of the ipsilateral anterior temporalis and masseter muscles; however, it would gradual improved to preoperative value in 12 months, which indicated masticatory weakness is only a temporary symptom after RFT. Corneitis is an especially important risk; we tested corneal reflection for each patient after RFT. If there is impaired corneal reflection or other uncomfortable symptoms of eyes, the patients require ophthalmology consultation immediately. We proposal artificial tears is a good medication for this complication, except 1 patient ignored our advice and lose her right eye sight eventually. Diplopia is a complication when abducens nerve was damaged by the RFT procedure, 2 patients were left with permanent diplopia induced by abducens nerve palsy. Corneitis and Diplopia maybe related with the cornea nerve (originated from V1 branch) and abducens nerve were injured in procedure. Because the V1 branch and abducens nerve is located in the deep and rear of Semilunar ganglion, respectively, when V1 involvement, the needle need puncture to the deeper position through foramen oval than V2 or V3 involvement, so the cornea nerve and abducens nerve more easily injured. Corneitis and Diplopia were all found in patients with V1 involvement also confirms this. Intracranial hypotension headache intracranial hypotension headache could be occurred when the needle puncture the wall of Meckel's cave. It is usually happened when the needle inserted to oval foramen, cerebrospinal fluid could be drained out of the hole on the wall of Meckel's cave. The patients should lie in bed absolutely for 2 days to prevent intracranial hypotension headache induced by cerebrospinal fluid outflow, because 2 patients suffered from this symptom who stood up 1 day after operation. After 2 days lie in bed absolutely after surgery, no one has intracranial hypotension headache occurred. There were no mortalities or life-threatening morbidities observed during or after RFT.

## CONCLUSION

CT guided RFT is a safe and effective procedure for ITN patients. It is an attractive treatment can give eligible patients a high success rate with less invasive. From our data, all different branches division of trigeminal neuralgia achieved comparable satisfactory curative effect; V2 obtained the best excellent pain relief, after RFT procedure. Facial numbness should be considered as an expected side effect rather than an unexpected complication, if prolonged freedom from pain is to be achieved. Patients who have pain in all 3 trigeminal divisions and patients who desire no facial numbness should be cautious. Masticatory weakness is mainly related with V3 branch RFT, while Corneitis and Diplopia with V1 branch RFT.

## References

[1] Koopman JS Dieleman JP Huygen FJ Incidence of facial pain in the general population. *Pain* 2009; 147:122–127. 19783099 10.1016/j.pain.2009.08.023

[2] Meyer B Lehmberg J Treatment options for refractory trigeminal neuralgia. *World Neurosurg* 2012; 77:275–276. 22120317 10.1016/j.wneu.2011.08.001

[3] Cruccu G Gronseth G Alksne J AAN-EFNS guidelines on trigeminal neuralgia management. *Eur J Neurol* 2008; 15:1013–1028. 18721143 10.1111/j.1468-1331.2008.02185.x

[4] Obermann M Treatment options in trigeminal neuralgia. *Ther Adv Neurol Disord* 2010; 3:107–115. 21179603 10.1177/1756285609359317 PMC3002644

[5] Emril DR Ho KY Treatment of trigeminal neuralgia: role of radiofrequency ablation. *J Pain Res* 2010; 3:249–254. 21311718 10.2147/JPR.S14455 PMC3033033

[6] Lopez BC Hamlyn PJ Zakrzewska JM Systematic review of ablative neurosurgical techniques for the treatment of trigeminal neuralgia. *Neurosurgery* 2004; 54:973–982.982–983. 15046666 10.1227/01.neu.0000114867.98896.f0

[7] Taha JM Tew JJ Comparison of surgical treatments for trigeminal neuralgia: reevaluation of radiofrequency rhizotomy. *Neurosurgery* 1996; 38:865–871. 8727810 10.1097/00006123-199605000-00001

[8] Tang YZ Jin D Li XY Repeated CT-guided percutaneous radiofrequency thermocoagulation for recurrent trigeminal neuralgia. *Eur Neurol* 2014; 72:54–59. 24853911 10.1159/000357868

[9] Tang YZ Jin D Bian JJ Long-term outcome of computed tomography-guided percutaneous radiofrequency thermocoagulation for classic trigeminal neuralgia patients older than 70 years. *J Craniofac Surg* 2014; 25:1292–1295. 25006910 10.1097/SCS.0000000000000591

[10] Sweet WH Wepsic JG Controlled thermocoagulation of trigeminal ganglion and rootlets for differential destruction of pain fibers. 1. Trigeminal neuralgia. *J Neurosurg* 1974; 40:143–156. 4587949 10.3171/jns.1974.40.2.0143

[11] Mittal B Thomas DG Controlled thermocoagulation in trigeminal neuralgia. *J Neurol Neurosurg Psychiatry* 1986; 49:932–936. 3746327 10.1136/jnnp.49.8.932 PMC1028956

[12] Tew JJ Keller JT The treatment of trigeminal neuralgia by percutaneous radiofrequency technique. *Clin Neurosurg* 1977; 24:557–578. 583707 10.1093/neurosurgery/24.cn_suppl_1.557

[13] Koopman JS de Vries LM Dieleman JP A nationwide study of three invasive treatments for trigeminal neuralgia. *Pain* 2011; 152:507–513. 21239113 10.1016/j.pain.2010.10.049

[14] Barker FN Jannetta PJ Bissonette DJ The long-term outcome of microvascular decompression for trigeminal neuralgia. *N Engl J Med* 1996; 334:1077–1083. 8598865 10.1056/NEJM199604253341701

[15] Mendoza N Illingworth RD Trigeminal neuralgia treated by microvascular decompression: a long-term follow-up study. *Br J Neurosurg* 1995; 9:13–19. 7786420

[16] Broggi G Franzini A Giorgi C Trigeminal neuralgia: new surgical strategies. *Acta Neurochir Suppl (Wien)* 1993; 58:171–173. 8109285 10.1007/978-3-7091-9297-9_40

[17] Kanpolat Y Savas A Bekar A Percutaneous controlled radiofrequency trigeminal rhizotomy for the treatment of idiopathic trigeminal neuralgia: 25-year experience with 1,600 patients. *Neurosurgery* 2001; 48:524–532.532–534. 11270542 10.1097/00006123-200103000-00013

[18] Hakanson S Trigeminal neuralgia treated by the injection of glycerol into the trigeminal cistern. *Neurosurgery* 1981; 9:638–646. 7322329 10.1227/00006123-198112000-00005

[19] Bale RJ Laimer I Martin A Frameless stereotactic cannulation of the foramen ovale for ablative treatment of trigeminal neuralgia. *Neurosurgery* 2006; 59 Suppl 2:S394–S401.S402. 10.1227/01.NEU.0000232770.97616.D0 17041509

[20] Sekimoto K Koizuka S Saito S Thermogangliolysis of the Gasserian ganglion under computed tomography fluoroscopy. *J Anesth* 2005; 19:177–179. 15875140 10.1007/s00540-005-0307-3

[21] Son BC Kim HS Kim IS Percutaneous radiofrequency thermocoagulation under fluoroscopic image-guidance for idiopathic trigeminal neuralgia. *J Korean Neurosurg Soc* 2011; 50:446–452. 22259692 10.3340/jkns.2011.50.5.446 PMC3259465

[22] Zheng S Wu B Zhao Y Masticatory muscles dysfunction after CT-guided percutaneous trigeminal radiofrequency thermocoagulation for trigeminal neuralgia: a detailed analysis. *Pain Pract* 2014; [Epub ahead of print]. 10.1111/papr.12247 25271538

